# Change in Threads on Twitter Regarding Influenza, Vaccines, and Vaccination During the COVID-19 Pandemic: Artificial Intelligence–Based Infodemiology Study

**DOI:** 10.2196/31983

**Published:** 2021-10-14

**Authors:** Arriel Benis, Anat Chatsubi, Eugene Levner, Shai Ashkenazi

**Affiliations:** 1 Faculty of Industrial Engineering and Technology Management Holon Institute of Technology Holon Israel; 2 Faculty of Digital Technologies in Medicine Holon Institute of Technology Holon Israel; 3 Faculty of Sciences Holon Institute of Technology Holon Israel; 4 Adelson School of Medicine Ariel University Ariel Israel

**Keywords:** influenza, vaccines, vaccination, social media, social networks, health communication, artificial intelligence, machine learning, text mining, infodemiology, COVID-19, SARS-CoV-2

## Abstract

**Background:**

Discussions of health issues on social media are a crucial information source reflecting real-world responses regarding events and opinions. They are often important in public health care, since these are influencing pathways that affect vaccination decision-making by hesitant individuals. Artificial intelligence methodologies based on internet search engine queries have been suggested to detect disease outbreaks and population behavior. Among social media, Twitter is a common platform of choice to search and share opinions and (mis)information about health care issues, including vaccination and vaccines.

**Objective:**

Our primary objective was to support the design and implementation of future eHealth strategies and interventions on social media to increase the quality of targeted communication campaigns and therefore increase influenza vaccination rates. Our goal was to define an artificial intelligence–based approach to elucidate how threads in Twitter on influenza vaccination changed during the COVID-19 pandemic. Such findings may support adapted vaccination campaigns and could be generalized to other health-related mass communications.

**Methods:**

The study comprised the following 5 stages: (1) collecting tweets from Twitter related to influenza, vaccines, and vaccination in the United States; (2) data cleansing and storage using machine learning techniques; (3) identifying terms, hashtags, and topics related to influenza, vaccines, and vaccination; (4) building a dynamic folksonomy of the previously defined vocabulary (terms and topics) to support the understanding of its trends; and (5) labeling and evaluating the folksonomy.

**Results:**

We collected and analyzed 2,782,720 tweets of 420,617 unique users between December 30, 2019, and April 30, 2021. These tweets were in English, were from the United States, and included at least one of the following terms: “flu,” “influenza,” “vaccination,” “vaccine,” and “vaxx.” We noticed that the prevalence of the terms vaccine and vaccination increased over 2020, and that “flu” and “covid” occurrences were inversely correlated as “flu” disappeared over time from the tweets. By combining word embedding and clustering, we then identified a folksonomy built around the following 3 topics dominating the content of the collected tweets: “health and medicine (biological and clinical aspects),” “protection and responsibility,” and “politics.” By analyzing terms frequently appearing together, we noticed that the tweets were related mainly to COVID-19 pandemic events.

**Conclusions:**

This study focused initially on vaccination against influenza and moved to vaccination against COVID-19. Infoveillance supported by machine learning on Twitter and other social media about topics related to vaccines and vaccination against communicable diseases and their trends can lead to the design of personalized messages encouraging targeted subpopulations’ engagement in vaccination. A greater likelihood that a targeted population receives a personalized message is associated with higher response, engagement, and proactiveness of the target population for the vaccination process.

## Introduction

### Background

As online-mediated communication environments increase, social media platforms enable individuals to discuss diverse issues, express their thoughts, and debate [1-3]. Twitter is a leading social network that provides microblogging services. Users can publish posts, called tweets, with a limited length of 280 characters. Thereby, users can interact with others by responding, sharing, or showing their interest by “liking” a tweet. These interactive abilities are the fundamental building blocks of the connective nature of social networks and serve as an echo of ideas transferred among users on the platform around the globe [4]. Retrieving information in tweets’ contents is challenging but is more manageable than in other social media platforms with long messages [5]. Indeed, the amount of structured and unstructured data from social media and Twitter has been increasing exponentially over the years [6,7]. Data mining and text mining enable the discovery of potentially new knowledge and contribute to developing efficient evidence-based decision-making tools [8-10] by extracting meaningful summaries, such as statistical ones, or controlled vocabularies (eg, terminology, folksonomy, taxonomy, and ontology) [11-15].

One of the most critical achievements of modern medicine is the development and widespread use of safe and efficacious vaccines. Nevertheless, their partial acceptance due to vaccine hesitancy and refusal is a significant health threat. Regarding influenza, compliance with the vaccine against it is relatively low compared with other vaccines, mainly because vaccination must be repeated annually [16]. Like other vaccines, influenza generates discussions both in the real world and online [17-20]. The COVID-19 vaccine is no exception.

Moreover, the global spread of the COVID-19 epidemic [21], its significant impact on daily life, and the relatively fast development of a vaccine against it have made the COVID-19 vaccine a critical health topic of discussion on social media. Reducing the incidence of transmissible diseases, such as influenza and COVID-19, requires achieving herd immunity [22,23], preferably by vaccination. This public health objective is achievable only with population engagement [18,19].

Social media platforms, such as Twitter, are a place of choice to share opinions and to search for (mis)information [24,25] about health care issues [26,27], including vaccines [17,18,28]. These open forums can influence opinions and vaccination decisions by hesitant individuals [29]. Discussions between provaccine advocates and “anti-vaxx” militants about vaccines’ necessity, effectiveness, and safety are continuous. Moreover, the internet as a whole enables the detection of early warnings of disease outbreaks, their dissemination tracking and resilience [30], and the spread of evidence-based information [31,32]. Artificial intelligence methods and algorithms (ie, data mining, text mining, and natural language processing) have been efficiently used in the last decade to detect outbreaks, such as influenza, based on emerging trends in internet search engine queries and social media threads [33-36]. There is a need for public health interventions [37] to make drastic stands against the spread of misinformation like that disseminated by vaccine opponents [19,38]. Related tools should be based on artificial intelligence to analyze efficiently and in an automated manner the big data generated over social media [39,40].

Understanding the changes happening during some health-related event discussions is crucial to improving health communication efficiency [41,42]. Disease prevention programs need to incorporate methods to make evidence-based information accessible to widespread populations using online resources and to increase control of biased and misleading announcements. The main focus is on advertising policies and campaigns on social media [30,43].

### Aims, Objectives, and Hypotheses

Our primary objective was to support the design and implementation of future eHealth strategies and interventions on social media to increase the quality of targeted communication campaigns and therefore increase influenza vaccination rates [18,19,44,45].

Our main aim was to define an artificial intelligence–based approach to analyze tweets, including terms related to vaccination against influenza and COVID-19. We focused on detecting co-occurring terms related to influenza vaccination and highlighting the dominant topics related to these terms. Therefore, these results must be used to build a folksonomy [46-49], which may then support the enhancement of vaccination campaigns. The methodology could be generalized to other health-related mass communications. Our research goal was to build a timely and dynamic vocabulary of the various topics related to influenza, vaccines, and vaccination posted in the English language. This vocabulary can be used as a decision support tool for health communication specialists and health policymakers, facilitating the understanding of the variations over time of different topics, such as those suggested in this study (tweets related to “influenza,” “vaccines,” and “vaccination”).

The following 4 hypotheses guided this research:

1. Tweets are a source of understanding the reasoning to take a vaccine.
2. “Influenza,” “vaccines,” and “vaccination” topics are not linked directly to other topics (such as politics, economics, and fears) but are related to health matters.
3. Actuality and news impact tweet content related to vaccines and vaccination.
4. The terms and hashtags of tweets about influenza, vaccines, and vaccination can be organized in a dynamic vocabulary [50]. It can reflect the main topics and their terms discussed over time on the social media platform.

This research was granted ethical approval by the Ethics Committee of the Faculty of Technology Management of the Holon Institute of Technology (Israel) (TM/2/2020/AB/004). The information collected on Twitter during this research was stored in a secured encrypted manner, with restricted access provided by the institution to the principal researcher (AB).

## Methods

### Overview

This study included the following 5 stages:

1. data sourcing to collect tweets and related data using the Twitter streaming application programming interface (API) [51];
2. data cleansing and storage;
3. identifying the terms, hashtags, and topics related to “influenza,” “vaccines,” and “vaccination;”
4. building a dynamic vocabulary, a folksonomy, to support the understanding of the relations between them; and
5. evaluating the vocabulary clusters.

### Data Sourcing

We extracted and collected tweets via the Twitter API for 16 months, between December 30, 2019, and April 30, 2021. These tweets were in English, from North America, and included at least one of the following terms: “flu,” “vaccination,” “vaccine,” and “vaxx” (this last term was used to capture messages related to vaccination opponents as these individuals use it). We selected these terms to maximize the chance to retrieve discussions concerning a vaccine as a product, vaccination as an act or a policy, vaccination hesitancy, and influenza. Moreover, since Twitter participants use informal language, for extracting influenza-related content, we used the popular term “flu.” The extraction omitted retweets and likes. The 16-month follow-up period allowed us to capture terms and topics of Twitter threads related to influenza, vaccines, and vaccination. Indeed, in the United States, 2020 involved the COVID-19 pandemic and the presidential elections.

### Data Preprocessing and Cleansing

To ensure efficient use of machine learning methods [52] on the tweet collection [53], we preprocessed it by cleansing and lemmatizing similar words appearing in posts. Data cleansing consisted of removing punctuation marks [54], mentions of users, glyphs, website addresses, and stop words [55]. Moreover, as the tweets were written in a natural language and concise manner (due to the limitation of 280 characters), a word may be written in several ways due to various reasons (eg, typos and short forms), all of which have the same or similar meaning. Lemmatization is one of the methods for overcoming this issue. It consists of replacing words by their root form (eg, “vaccine” for “vaccines”). [56]. For example, due to the COVID-19 pandemic, the tweets retrieved during the collection process contained multiple representations of the term “covid,” such as “COVID19,” “COVID-19,” and “coronavirus.” We used the Python Natural Language Toolkit (NLTK) package for lemmatization [55]. Since the nature of tweets is informal, it has been assumed that using a single representation of those words will not significantly change the tweet’s context, thus improving the model’s accuracy. Therefore, the frequent representations of the term “COVID” were replaced with the single form “covid,” and the terms related to “influenza” were lemmatized to “flu” as the popular language used on Twitter. All the lemmas were stored in lowercase.

### Identifying the Terms and Topics Related to Influenza, Vaccines, and Vaccination

We handled the identification of the terms, hashtags, and topics related to influenza, vaccines, and vaccination by a 3-step process as follows: (1) clustering with word embedding and n-grams, (2) building a folksonomy, and (3) evaluating folksonomy clusters.

#### Clustering

The objective of clustering is to segregate a set of points into groups, with each one as similar as possible and different from the others [57]. For example, in the context of text mining and specifically mining a tweet corpus, clustering can be used to group terms that are semantically similar or frequently appearing in the same message. Each cluster, according to its content, can then be annotated with a topic.

##### Word Embedding

Handling the high volume of collected tweets over time means dealing with the curse of dimensionality [58]. Therefore, a symbolic-numeric reformulation associated with dimension reduction [59] must be used to handle a large amount of data in a reasonable time and reduce the processing complexity. Word embedding is a relevant approach supporting these 2 goals; it consists of a learned numerical representation of text where words having a similar meaning in a specific context have an equal numerical representation in a vector. Globally, word embedding allows the prediction of words in a specific context. Thus, Word2Vec is a word embedding algorithm based on a neural network model learning from a large corpus of text (ie, a context) the association between words or terms. After the first training step, Word2Vec can detect synonymous words or terms, or suggest complete sentences. This is done by searching for vectors and so words with a close semantic similarity represented by cosine or Euclidean distance (ie, the similarity or the relation) between two vectors (ie, words and terms) in a space (ie, corpus) of *n* dimensions (ie, number of words or terms in the corpus) [60]. As an example, words related to time, such as “day,” “week,” “month,” “season,” and “year,” will be used in similar contexts and will be defined as semantically closed. The preprocessed data were used for creating the Gensim Word2Vec model in Python [61]. In order to see each word in the context it has with other words, we produced clusters, with the K-means algorithm [62,63], to assist decision makers in better understanding the public’s perceptions of vaccines and vaccination against influenza and COVID-19. As discussions constantly evolve, the word embedding and clustering process was repeated monthly on newly collected tweets.

##### N-grams

As a complementary approach to word embedding, we built an n-gram language model predicting the probability of a sequence of words (after stop-word cleaning) to appear in our corpus of tweets. We extracted the most frequent n-grams comprising between 1 and 4 terms (n) for each week. Moreover, this process used the Gensim Python library [61]. This approach enables health communication decision makers to learn about new growing or shrinking isolated terms and sets of terms in the discussions related to vaccination and influenza.

##### Defining the Numbers of Clusters as Topics

Clustering is an unsupervised learning task and is challenging due to the need to define *k* and the number of clusters to build. The “silhouette method” allows assessing the quality of clustering, as it determines the similarity of an object (eg, a word also called a unigram) with the content of its cluster and the likeness with the other clusters. A silhouette shows which objects (eg, words, vectors, and values) lie well within a cluster and which are less related. The graphical combination of the silhouettes of an entire clustering (eg, with *k* clusters) into a single plot allows the appreciation of each cluster’s relative quality and the overall clustering itself. The overall average silhouette width (ie, the average silhouette width of each cluster) provides an evaluation of clustering validity. A higher value of the overall average silhouette width (ie, silhouette score) is associated with better clustering with *k*, and therefore, it must be selected as the better partitioning. The silhouette method is independent of the partitioning algorithm used [64]. From our research perspective, each term must have a minimum number of occurrences to be included in the analysis. Moreover, 2 terms must have a maximum distance (number of other terms) between them in a tweet to consider their potential semantic link.

##### Cluster Visualization

Cluster visualization is produced by using t-distributed stochastic neighbor embedding (t-SNE), which is a nonlinear dimensionality reduction technique for embedding high-dimensional data and visualizing it in a low-dimensional (ie, 2 or 3) space [65].

##### Evaluation of the Terms in the Clusters and as N-grams

To evaluate our approach and the results of identifying the terms, hashtags, and topics related to influenza, vaccines, and vaccination, we implemented a validation process built on complementary approaches. One focused on the word embedding results, the second focused on n-grams, and the third focused on the whole by involving social media users. Thus, the terms were grouped once from a semantic perspective with word embedding on the first hand and once from a high coappearance frequency as n-grams describe the content of the explored Twitter threads in summarized ways.

The second evaluation approach consisted of using Google Trends [66] for getting the relative frequency of search terms during a specific period and in a specific geographic area. In this study, the n-grams (n between 1 to 4) were extracted from the tweets, and their weekly frequency was calculated. Next, the n-grams that appeared in the top 150 list continuously for at least 12 weeks were used as an input for a Google Trends query at the time frame they were published on Twitter. Finally, the n-grams (bi-grams) and the Google Trends query results were normalized. Their Pearson correlation coefficients were calculated by considering the weekly tweet-based n-grams and the weekly relative number of queries (comprising the n-gram terms) on the Google search engine.

The third evaluation consisted of computing Pearson correlations between the weekly frequency (between December 2020 and April 2021) of n-grams specific to vaccines, vaccination, influenza, and COVID-19, and the proportion of the population vaccinated against COVID-19.

### Informed Consent Statement

The social network data were collected in an anonymized way and following Twitter’s rules. The participants of the evaluation survey provided anonymous informed consent in an electronic way on the platform before they could proceed to the completion of the questionnaire.

### Data Availability Statement

The Twitter data that support the findings of this study are not available owing to Twitter’s rules and regulations. The survey data that support the findings are available from the corresponding author (AB) upon reasonable request, which will need to undergo ethical and legal approvals by the investigators’ institutions. The methodology of this research will be reported in the AIMe registry for artificial intelligence in biomedical research [67].

## Results

### Descriptive Statistics

**Figure 1:**
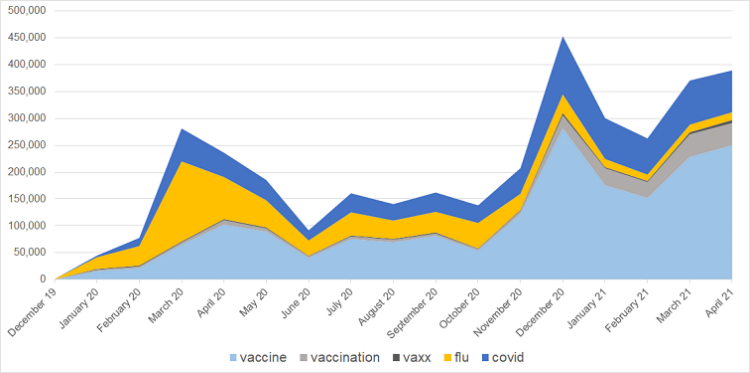
Distribution of the number of tweets by month comprising at least one of the terms “flu,” “vaccination,” “vaccine,” “vaxx,” and “covid” between December 30, 2019, and April 30, 2021.

A total of 2,782,720 tweets of 420,617 unique users between December 30, 2019, and April 30, 2021, were collected. The graph in Figure 1 shows the number of tweets per month (bar columns) containing at least one of the following terms (or similar after cleansing and lemmatization): (1) “flu,” (2) “vaccination,” (3) “vaccine,” (4) “vaxx,” and (5) “covid.” The lines in Figure 1 show the proportion in percentage of each of these terms in the collected tweets. Although the term “covid” and its synonyms were not part of the initial keywords used for querying tweets, its emergence reflects the effect of the COVID-19 pandemic as an important topic in the discussions regarding vaccination and influenza in 2020 and 2021.

Figure 1 also shows that globally the number of tweets comprising at least one of the terms “flu,” “vaccination,” “vaccine,” “vaxx,” and “covid” has dramatically increased over the period from December 2020 to April 2021 (see also Multimedia Appendix 1). Two peaks were noticed. The first was in March 2020, with the World Health Organization declaring COVID-19 as a pandemic (March 11, 2020) and President Donald Trump promulgating COVID-19 as a national emergency (March 13, 2020). The second peak in December 2020 was related mainly to “vaccines” in response to the approval of COVID-19 vaccines (Food and Drug Administration [FDA] emergency use authorizations for Pfizer BioNTech vaccine on December 11, 2020, and Moderna vaccine on December 18, 2020). Thus, the term “vaccine” increased from approximately 35% in January 2020 to approximately 80% one year later. In contrast, the term “vaxx” (for the terms “antivaxx,” “antivaxxer,” “anti-vaxx,” and “anti-vaxxer”) was stable at 1% to 3% over the whole data collection period. Nevertheless, it is essential to take into account that vaccination opponents used various tools and communication discourse, not evocating the “anti-vaxx” term itself [68-70]. The terms related to influenza (“flu”) and COVID-19 (“covid”) showed an inverse correlation (*r*=−0.83, *P*<.001) at the monthly level (Multimedia Appendix 1). The use of “covid” increased linearly, starting in January 2020, with the first cases of COVID-19 spreading from China to Europe and the United States [71], until February 2021, when it was part of approximately 35% of the collected tweets. In parallel, the use of the term “flu” decreased steadily, probably due to the low influenza activity during the 2020-2021 season [72,73].

### Identification of the Terms and Topics Related to Influenza, Vaccines, and Vaccination

#### Word Embedding

The Word2Vec algorithm was run monthly to find the optimal parameters supporting the finding of the dominant trending topics. Determination of the optimal parameters’ values was performed by creating models using a different value for each parameter and calculating the silhouette score for each iteration with the “silhouette_score” function of sklearn.metrics in Python [74]. Multimedia Appendix 2 shows the parameters’ values and the silhouette scores of the various models of each month. Moreover, each week, only the terms having the highest occurrence regarding the overall number of terms detected in the tweets collected in the same week were investigated. The values of these attributes were changed over time to consider the dynamic changes in social media users’ lexicons impacted by the actuality.

#### K-means Clustering

Using the monthly word embedding model as an input, word clusters were generated with the NLTK KMeansClusterer [75]. The clustering method groups together a given data set to a *k* predetermined number of clusters [66,76]. The partition is performed while aiming to minimize the in-cluster variance and maximize the variance between the elements from different clusters. To determine the optimal number of clusters [77], we computed the silhouette scores of k-means clustering runs with *k* ∈ [3;6]. The silhouette scores of the clustering models were generated on the 2,782,720 tweets of 420,617 unique users between December 30, 2019, and April 30, 2021, related to 141,407 n-grams with *n* ∈ [2;4]. The highest silhouette score reflects this grouping, wherein the different objects are well affected to their clusters and less linked to neighboring and less relevant clusters. A higher silhouette score (*s*=0.72) was achieved with *k*=3. This score can be considered good as we clustered terms that can relate to different topics and the clusters can overlap partially [78,79]. Furthermore, by computing the Ray-Turi index [80] for *k* between 2 and 10, and building the curve of the different generated values allowed with the Elbow method, the optimal *k* was equal to 3 [81].

Indeed, we interpreted the content of the 3 clusters in the tweet collection of the study with consensus of domain experts (public health, infectiology, and informatics). These clusters are the bare bricks of the “vaccination against influenza during the COVID-19 pandemic” folksonomy. We defined the 3 topics dominating the content of the collected tweets as follows:

1. “Health and medicine (biological and clinical aspects)” comprising terms such as “pandemic,” “COVID-19,” “vaccines,” “illness,” “die,” “variant,” “children,” “flu,” “influenza,” and “health;”
2. “Protection and responsibility” with terms such as “protection,” “social distancing,” “vaccination,” “fighting COVID-19,” and “responsibility;” and
3. “Politics” supported by terms like “trump,” “biden,” “lie,” “government,” “trust,” “bill_gate,” “free,” “money,” “president,” “politics,” “politicians,” “elections,” “vaccine,” and “policy.”

**Figure 2:**
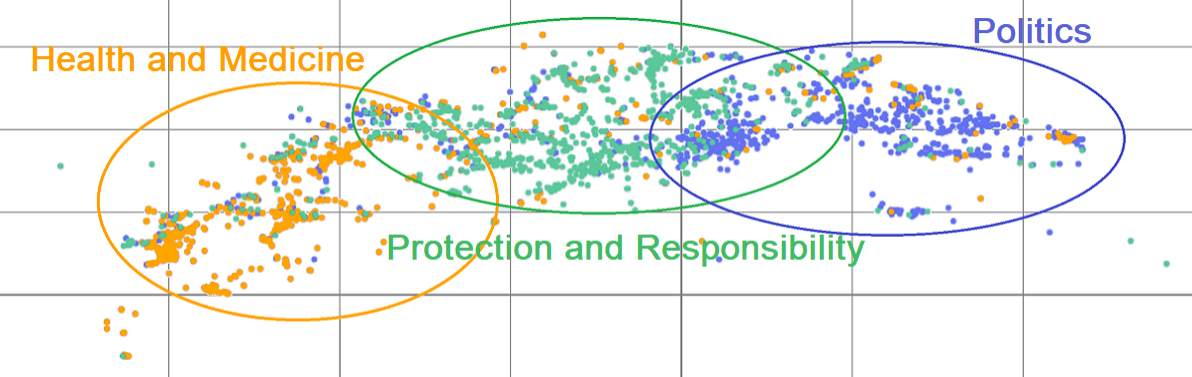
A t-distributed stochastic neighbor embedding graphical representation of the 3 topic clusters with 1000 most frequent n-grams (*n* ∈ [1;4]). Orange, seafoam (green-blue facilitating reading of the figure by color-blind individuals), and violet represent “health and medicine (biological and clinical aspects),” “protection and responsibility,” and “politics,” respectively.

Figure 2 shows a 2-dimensional graphical representation of the 3 clusters with the 1000 most frequent n-grams for each (*n* ∈ [1;4]) (Multimedia Appendix 3 and Multimedia Appendix 4), which has been generated by using the t-SNE algorithm [65].

Explicitly, this visualization (Figure 2) allows us to see the first 1000 most used terms in the tweets of each one of the previously computed clusters. It is noticeable that overlaps exist between the clusters, which is quite logical when we realize that the tweets relate in many cases to a few topics at the same time (eg, from an account dealing with *political* issues: “The *vaccines* offer good *protection* with more than 80% effectiveness. Most people will not be *sick* and the ones that will, will not get seriously *ill* or *die*”).

#### N-grams

The preprocessed tweets were used to extract n-grams for each week. Multimedia Appendix 4 shows the 10 most common n-grams for each *n* ∈ [1;4]. For example, the words “flu” and “bad” were found close to each other in the word embedding model over the months of this study (Multimedia Appendix 3, list of the 1000 most frequent n-grams for cluster 1). Those 2 words were also a common n-gram, whether a bigram or a part of a higher degree of an n-gram. Although included in the word embedding representation, we see the relations between those 2 words in general, as they get closer to each other and in the same semantic cluster.

Following the extraction, each n-gram received its growth value, indicating an increased or decreased n-gram frequency from the previous week. The growth is used to highlight the significant changes in the n-grams and therefore in general discussions. For example, on November 9, 2020, Pfizer BioNTech published the initial results of the COVID-19 vaccine trial, which showed high efficacy against the disease. The n-grams of the same week showed a significant increase as follows: “*take, vaccine*,” 774.6% (1207/51,553 vs 138/51,553) and “*get, vaccine*,” 557.9% (1987/149,333 vs 302/149,333) [82].

Moreover, in mid-March 2021, we also noticed a significant increase in n-grams related to vaccination against COVID-19 due to reports on Twitter of individuals being vaccinated or local authorities inviting the population to schedule appointments for taking the vaccine (eg, “vaccine, appointment, available,” +264.9% [748/18,678 in the week starting March 15, 2021, vs 205/18,678 in the week starting March 08, 2021] and “code, vaccine, appointment, available,” +251.5% [942/6264 in the week starting March 29, 2021, vs 268/6264 in the week starting March 22, 2021]) [83].

Another example of Twitter’s user response was during the week starting May 11, 2020. The leading n-grams were “*social distancing, flattenthecurve, trump, test*” and “*flattenthecurve, trump, test, vaccine*” (wherein “socialdistancing” and “flattenthecurve” were hashtags). Both demonstrated growth of 693.0% from the previous week (43 occurrences during the week starting May 04, 2020, vs 341 out of 516 occurrences in total). In that week, Forbes magazine published an article reporting that hospitals across the United States are “*not being overwhelmed*,” suggesting that the efforts for flattening the curve have succeeded. The overall results show how the tweet threads about influenza, vaccines, vaccination, and COVID-19 dynamically evolved from the end of 2019 to mid-2021.

### Evaluations

#### Google Trends Validation

As a component of the internet, social media like Twitter are a part of how people get and share information and knowledge. Therefore, looking at queries on search engines like Google allows the evaluation of global interests in terms and topics detected on social media. Thus, we computed Pearson correlations between the weekly occurrences of n-grams in tweets and weekly queries in the Google search engine and those reported on Google Trends [84]. As an example of the consistency of the previously disclosed results, the n-gram of “flu, symptom” on Twitter and the number of queries on Google were highly correlated (*r*=0.85, *P*<.001) between January 1, 2020, and March 4, 2021 (Table 1). During these 65 weeks, this n-gram (ie, “flu, symptom”) was also used to search for information about “influenza” and “symptoms.”

Moreover, as we noticed the decreasing popularity of its use on Twitter, we also noticed similar behavior on Google. Additionally, the n-gram “covid, vaccine” also showed a high correlation between Twitter and Google (*r*=0.85, *P*<.001), and on the 2 platforms, its occurrence increased between January 2020 and January 2021, and then showed a parallel decrease. Globally, the top topics related to vaccines, vaccination, and COVID-19 were similar on social networks and search engines (Table 1). Thus, internet users’ queries on search engines relate with the timing of topics defined by analysis of the text of our Twitter message data set.

**Table 1:** Examples of n-grams having high correlations between their trend frequencies in tweets and Google search queries.

N-gram	Period (start date to end date)	Pearson correlation	*P* value
get, second, dose	January 04, 2021, to April 30, 2021	0.91	<.001
get, first, vaccine, shot	January 18, 2021, to April 25, 2021	0.89	<.001
second, vaccine	February 01, 2021, to April 30, 2021	0.86	<.001
flu, symptom	January 01, 2020, to April 04, 2021	0.85	<.001
covid, vaccine	January 20, 2020, to April 30, 2021	0.85	<.001
think, flu	January 01, 2020, to March 30, 2020	0.84	<.001
second, dose, vaccine	January 04, 2021, to April 30, 2021	0.84	<.001
get, second, vaccine	February 01, 2021, to April 30, 2021	0.84	<.001
get, covid, vaccine	March 30, 2020, to April 30, 2021	0.84	<.001
get, vaccine	January 01, 2020, to April 30, 2021	0.80	<.001

#### Real-World Validation

On December 11, 2020, the FDA issued an emergency use authorization for a COVID-19 vaccine. A few days later, on December 20, 2020, vaccination of the population with the Pfizer BioNTech vaccine was started. We downloaded the daily vaccination rate from Centers for Disease Control and Prevention (CDC) publications and aggregated them at the weekly level [85]. We noticed that starting in December 2020 and ending on April 30, 2021, Pearson correlations between the weekly occurrences of COVID-19 vaccination n-grams and the weekly vaccination rates (Table 2) were high and significant (*r*>0.81, *P*<.001) [86]. These results demonstrate that the tweets of this study mirror “real-life” significant events during the pandemic.

**Table 2:** Correlations of the 5 highest n-gram trends with the vaccination rate trends reported by the Centers for Disease Control and Prevention between December 20, 2020, and April 30, 2021.

N-gram	Pearson correlation	Number of occurrences	*P* value
get, first	0.88	17,133	<.001
vaccine, today	0.87	9205	<.001
first, vaccine	0.83	9260	<.001
first, dose	0.82	11,357	<.001
vaccine, shot	0.81	11,113	<.001

## Discussion

### Principal Findings

This research was initiated to elucidate online public perceptions regarding vaccination, mainly against seasonal influenza. However, the COVID-19 pandemic in 2020 was impressively reflected by major changes in the focus of Twitter-based discussions. The most important aspect of this study is the building of a folksonomy based on tweet text analysis, word embedding, and clustering. The 3 topics that were identified in this folksonomy were as follows:

1. General issues from the “health and medicine (biological and clinical aspects)” perspective. The initial terms used for the tweet extraction were “flu,” “vaccination,” “vaccine,” and “vaxx.” These terms are de facto strongly related to health and medicine, and generate a large spectrum of threats (ie, from asking/answering questions about symptoms, reporting health conditions, and sharing positions). The presence of terms related to the COVID-19 pandemic is understandable given the period of the data collection.
2. “Protection and responsibility” as a central dimension of the decision to take a vaccine or not. The COVID-19 pandemic showed the need for social distancing and mask wearing to reduce the spread of the virus. For these reasons, tweets related to influenza (“flu”) or immunization (“vaccine” and “vaccination”) and, by extension, to COVID-19 comprise threads discussing protection measures (like vaccination) and the responsibility to use them (such as taking a vaccine). It is important to highlight, based on prior studies [19,87,88], that the intent to take a vaccine is considered by the younger adult US population as an act of collective responsibility.
3. “Politics” is a cluster showing the divergence of opinions and messages of US political leaders (ie, Republicans and Democrats) about the severity of the crisis and the efforts to reduce disease transmission [89]. Besides this cluster, it is important to remember that in parallel to the first year and first waves of the COVID-19 pandemic, 2020 was an election year. Thus, the local and national management of this global epidemic was a source of political debates, and support or criticism of governments, administrations, and the health care system.

The mechanisms behind the folksonomy rely on a complex set of factors. First, as pointed out above, the reasons for the emergence of each cluster depend on both culture and real-life events. Second, these mechanisms can be quantified by analyzing terms that frequently appear together (n-grams). Thus, in the context of this research, we observed that the main focus of the tweets related mainly to COVID-19 pandemic events (disease, confinement, politician talks, vaccines approval, and vaccination) and increased over time, like the prevalence of the terms “vaccines” and “vaccination,” and this was in contrast with the term “flu,” which disappeared over time from the tweets. This reflects that COVID-19 measures, such as social distancing and mask wearing, significantly reduced the seasonal influenza rates in 2020-2021 [73,90,91]. However, a potential major reason and mechanism of these changes in trends and therefore of the folksonomy content may be associated with the diversion of citizens’ attention to annual influenza spread, caused by the disruptive and menacing COVID-19 pandemic. These distractions induced different behaviors or feelings, such as devastation, fear, worry, and the need to understand [92,93].

### Strengths and Limitations

Social media and social networks are increasingly being used to disseminate multimodal and multisource-based health-related information in a timely manner. In the context of epidemics and pandemics, such as seasonal influenza and COVID-19, health care organizations and governmental institutions nowadays spread information and run communication campaigns on social media, for example, to increase citizen engagement in vaccination. At the same time, individuals share their positions, even if it is associated with the antivax trend, and sometimes spread misinformation [94]. The strength of our study is its ability to provide health authorities with a weekly, monthly, and long-term folksonomy of the emerging or persisting topics of social media threads related to a health care issue or event, such as vaccination or a virus-related matter. Providing a folksonomy and the co-occurring terms in the same or additional clusters, using these tools, can enhance health-related social media campaigns, focusing on grand public in-time interests and queries, similar to the approaches used in other business fields.

By getting reports in a timely manner, it has been proven possible to point out the various topics, words, and terms frequently used on social media, thereby enabling health communication specialists, and more specifically those dealing with social media, to focus on up-to-date campaigns to increase population engagement, such as that done in other business fields [95], and actions related to health promotion, especially during epidemics and crises [96] (eg, H1N1 [97] and Ebola [98]), as has been suggested in prior research not dealing with terms, topics, and target population discovery or designation [99].

Exploring social media, and more particularly social networks, is limited by the passive exclusion of nonusers of these communication channels or inactive users who only read posts but do not post by themselves or respond to the messages of other users.

Another limitation of this study is that it was based only on tweets in English and posted from North America. This filtering limits the generalization of the results. The diversity of the US population suggests that running this kind of study in the United States in other languages will enable fine-tuning of health communication and increase vaccination compliance in non-English speaking communities (ie, around 22.0% of the US population) [19,100].

In parallel with our study, another study dealing specifically and strictly with vaccination and COVID-19 was performed among Australian Twitter users (versus US Twitter users in our study) between January and October 2020 (versus between December 2019 and April 2021 in our study) and collected 31,100 tweets (versus 2,782,720 tweets collected by us). The analysis was based on latent Dirichlet allocation, which is an unsupervised learning approach that can be large-scale intensively system resource consuming [101]. The Australian tweet analysis revealed the following 3 dominant topics: (1) “COVID-19 and its vaccination,” (2) “advocacy for infection control measures and vaccine trials,” and (3) “conspiracy theories, complaints, and misinformation” [102]. Even though some convergence exists, these results are distinct from ours by focusing more specifically on COVID-19–related issues.

Moreover, the set of words initially used for extracting the tweets (“influenza” OR “vaccine” OR “vaccination” OR “vaxx”) allowed us to capture a larger spectrum of threads related to each one of the terms that we were interested in focusing on and not in a strict filtering approach, as in other prior research [101]. Nevertheless, without extending the extraction word set, with terms of the COVID-19 pandemic, tweets potentially interesting but not comprising one of these terms would not have been extracted. For example, the following tweet published in mid-April 2021 that included words detected in the n-gram analysis but not explicitly the words used for the tweet’s extraction failed to be retrieved: “I am excited, I am in my county seat to get my first injection of the Pfizer.” A future perspective for enhancing the dynamic of trend tracking can be considered to update the terms of the tweet extraction query with other disrupting terms due to actuality (eg, “covid,” “dose,” “injection,” and trade names of vaccines). This enhancement can be achieved by a domain expert (ie, human action) or by automatically selecting words emerging as trending in a cluster of the folksonomy and co-occurrence frequency analysis (ie, n-grams) [95].

Additionally, when dealing with the large volume of tweets generated each minute, looking at all tweets in real time is impossible without deploying a high computational infrastructure, which is available in dedicated centers. Accordingly, the objective of this research was to define a framework enabling health system decision makers to focus on specific issues in order to enhance their social media campaigns by understanding the topics discussed in a particular context (ie, vaccination and influenza). Furthermore, the tweets are collected daily (due to Twitter constraints, without using a paying platform) and analyzed, with the machine learning flow described in the methodology, at the weekly, monthly, and all-time levels. To deal with others’ terms of interest, changing the terms of the tweet extraction query will allow the expansion of the current data set or the start of new research with the same methodology. This study shows that combining social media data, such as tweets, and artificial intelligence approaches, such as machine learning algorithms for text and data mining, enables an infodemiology and infoveillance study as a whole. More specifically, in this study, we noticed the strength of this combined approach by following the changes in the contents and topics of the tweets over time and the influence of the actual events. Like other Twitter-based public health research, the approach of collecting, analyzing, and assessing in near real time the content of messages provides powerful indications to health decision makers for adapting and enhancing communication as an emergency response and in planning [103]. In other words, these forewarnings must support social media–based health information in targeting advertisements of recommendations, instructions, and directives, according to social media user’ interests and focuses (ie, terms appearing in the clusters of the folksonomy) disclosed passively in previous posts, shares, or likes. Moreover, social media platforms allow accurate targeting by stratifying advertising campaigns on sociodemographic attributes, such as age, gender, marital status, location, spoken language, and educational and professional background [104]. Thus, social media–based health information is intended to increase population adherence to health policies, such as vaccination against epidemic or pandemic diseases (eg, influenza and COVID-19), by delivering personalized messages taking into account both sociodemographics and domains of interest. For example, a young person playing basketball, living in an area with recurrent high acute influenza incidence in a young population, following social media groups dealing with basketball, and sharing posts related to vaccination hesitancy will get advertisements with personalized content targeting young vaccination-hesitant individuals playing collective sports and emphasizing that vaccination is the best solution to continue this activity during an epidemic [105].

### Conclusions

Twitter is one of the leading social network platforms allowing anyone to share positions and information in any domain. Therefore, any kind of information published and spread about influenza and COVID-19, and the vaccines against each, can be perceived as reliable and can influence social media users. Specifically, during the COVID-19 pandemic, world leaders have widely used Twitter to communicate public health information with citizens. These messages had a strong effect on vaccination compliance [106], with the ability to dynamically improve the content and target health communication campaigns on social media.

This study allowed us to validate our initial hypothesis. Tweets are a source of information for understanding why it is recommended to take a vaccine and the public perception about it [107-109]. Indeed, we defined a folksonomy of the 3 main topics coexisting in the collected messages over 16 months. Accordingly, the terms and hashtags of tweets concerning “influenza,” “vaccines,” and “vaccination” can be organized in a dynamic vocabulary, such as a folksonomy, reflecting the main topics and their terms discussed over time on the social media platform. Additionally, the emergence and dominance of terms related to COVID-19 over time, reported in the folksonomy with frequently co-occurring words, shows that although the study did not initially focus on this thematic, the health changes are reflected in the Twitter threads related to vaccines and vaccination.

This study focused initially on vaccination against influenza and moved to vaccination against COVID-19. Infoveillance on Twitter (and other social media) about the topics related to vaccines and vaccination against communicable diseases can create opportunities to design and convey personalized messages encouraging specific targeted subpopulations’ engagement in vaccination. A greater likelihood that a targeted population receives a personalized message is associated with a higher response, engagement, and proactiveness of the target population for vaccination or other public health measures [110].

## Appendix

Multimedia Appendix 1 Number of tweets by month comprising at least one of the terms "flu," "vaccination," "vaccine," "vaxx," and "covid.".

Multimedia Appendix 2 Optimal number of clusters, the related maximal silhouette score for each month, and the parameters used for creating the topic clustering.

Multimedia Appendix 3 List of the 1000 most frequent n-grams in the 3 clusters.

Multimedia Appendix 4 N-grams having the highest increase in occurrence week over week.

## Abbreviations

API: application programming interface
FDA: Food and Drug Administration
NLTK: Natural Language Toolkit
t-SNE: t-distributed stochastic neighbor embedding

## References

[1] Bello-Orgaz G, Hernandez-Castro J, Camacho D (2017). Detecting discussion communities on vaccination in twitter. Future Generation Computer Systems.

[2] Grajales FJ, Sheps S, Ho K, Novak-Lauscher H, Eysenbach G (2014). Social media: a review and tutorial of applications in medicine and health care. J Med Internet Res.

[3] Choi S (2014). The Two-Step Flow of Communication in Twitter-Based Public Forums. Social Science Computer Review.

[4] Mosleh M, Pennycook G, Arechar AA, Rand DG (2021). Cognitive reflection correlates with behavior on Twitter. Nat Commun.

[5] Trye D, Calude AS, Bravo-Marquez F, Keegan TT (2020). Hybrid Hashtags: #YouKnowYoureAKiwiWhen Your Tweet Contains Māori and English. Front Artif Intell.

[6] Inmon WH (2015). Data Architecture: a Primer for the Data Scientist || The Data Infrastructure.

[7] Big Data. Gartner.

[8] Zhou S, Qiao Z, Du Q, Wang GA, Fan W, Yan X (2018). Measuring Customer Agility from Online Reviews Using Big Data Text Analytics. Journal of Management Information Systems.

[9] Abbas A, Zhou Y, Deng S, Zhang P (2018). Text Analytics to Support Sense-Making in Social Media: A Language-Action Perspective. MISQ.

[10] Hoogeveen D, Wang L, Baldwin T, Verspoor K (2018). Web Forum Retrieval and Text Analytics: A Survey. FNT in Information Retrieval.

[11] Mokkink LB, Terwee CB, Patrick DL, Alonso J, Stratford PW, Knol DL, Bouter LM, de Vet HCW (2010). The COSMIN study reached international consensus on taxonomy, terminology, and definitions of measurement properties for health-related patient-reported outcomes. J Clin Epidemiol.

[12] Vrijens B, De Geest S, Hughes DA, Przemyslaw K, Demonceau J, Ruppar T, Dobbels F, Fargher E, Morrison V, Lewek P, Matyjaszczyk M, Mshelia C, Clyne W, Aronson JK, Urquhart J, Project Team ABC (2012). A new taxonomy for describing and defining adherence to medications. Br J Clin Pharmacol.

[13] Šmite D, Wohlin C, Galviņa Z, Prikladnicki R (2012). An empirically based terminology and taxonomy for global software engineering. Empir Software Eng.

[14] Zimbra D, Abbasi A, Zeng D, Chen H (2018). The State-of-the-Art in Twitter Sentiment Analysis. ACM Trans. Manage. Inf. Syst.

[15] de Lusignan S, Liyanage H, McGagh D, Jani BD, Bauwens J, Byford R, Evans D, Fahey T, Greenhalgh T, Jones N, Mair FS, Okusi C, Parimalanathan V, Pell JP, Sherlock J, Tamburis O, Tripathy M, Ferreira F, Williams J, Hobbs FDR (2020). COVID-19 Surveillance in a Primary Care Sentinel Network: In-Pandemic Development of an Application Ontology. JMIR Public Health Surveill.

[16] Immunization coverage. World Health Organization.

[17] Ashkenazi S, Livni G, Klein A, Kremer N, Havlin A, Berkowitz O (2020). The relationship between parental source of information and knowledge about measles / measles vaccine and vaccine hesitancy. Vaccine.

[18] Benis A, Khodos A, Ran S, Levner E, Ashkenazi S (2021). Social Media Engagement and Influenza Vaccination During the COVID-19 Pandemic: Cross-sectional Survey Study. J Med Internet Res.

[19] Benis A, Seidmann A, Ashkenazi S (2021). Reasons for Taking the COVID-19 Vaccine by US Social Media Users. Vaccines (Basel).

[20] Deiner MS, Fathy C, Kim J, Niemeyer K, Ramirez D, Ackley SF, Liu F, Lietman TM, Porco TC (2019). Facebook and Twitter vaccine sentiment in response to measles outbreaks. Health Informatics J.

[21] WHO Coronavirus (COVID-19) Dashboard. World Health Organization.

[22] Randolph HE, Barreiro LB (2020). Herd Immunity: Understanding COVID-19. Immunity.

[23] McDermott A (2021). Core Concept: Herd immunity is an important-and often misunderstood-public health phenomenon. Proc Natl Acad Sci U S A.

[24] Rainie L, Wellman B (2014). Networked: The New Social Operating System.

[25] Wu L, Morstatter F, Carley KM, Liu H (2019). Misinformation in Social Media. SIGKDD Explor. Newsl.

[26] Eysenbach G (2002). Infodemiology: The epidemiology of (mis)information. Am J Med.

[27] Morley J, Cowls J, Taddeo M, Floridi L (2020). Public Health in the Information Age: Recognizing the Infosphere as a Social Determinant of Health. J Med Internet Res.

[28] Cordina M, Lauri MA, Lauri J (2021). Attitudes towards COVID-19 vaccination, vaccine hesitancy and intention to take the vaccine. Pharm Pract (Granada).

[29] MacDonald NE, SAGE Working Group on Vaccine Hesitancy (2015). Vaccine hesitancy: Definition, scope and determinants. Vaccine.

[30] Gupta A, Katarya R (2020). Social media based surveillance systems for healthcare using machine learning: A systematic review. J Biomed Inform.

[31] Thomson A, Vallée-Tourangeau G, Suggs LS (2018). Strategies to increase vaccine acceptance and uptake: From behavioral insights to context-specific, culturally-appropriate, evidence-based communications and interventions. Vaccine.

[32] Alessa A, Faezipour M (2018). A review of influenza detection and prediction through social networking sites. Theor Biol Med Model.

[33] Aramaki E, Maskawa S, Morita M (2011). Twitter catches the flu: detecting influenza epidemics using Twitter. EMNLP '11: Proceedings of the Conference on Empirical Methods in Natural Language Processing.

[34] Talvis K, Chorianopoulos K, Kermanidis K (2014). Real-Time Monitoring of Flu Epidemics through Linguistic and Statistical Analysis of Twitter Messages. https://ieeexplore.ieee.org/document/6978958.

[35] Wakamiya S, Kawai Y, Aramaki E (2018). Twitter-Based Influenza Detection After Flu Peak via Tweets With Indirect Information: Text Mining Study. JMIR Public Health Surveill.

[36] Hassan Zadeh A, Zolbanin HM, Sharda R, Delen D (2019). Social Media for Nowcasting Flu Activity: Spatio-Temporal Big Data Analysis. Inf Syst Front.

[37] Faasse K, Chatman CJ, Martin LR (2016). A comparison of language use in pro- and anti-vaccination comments in response to a high profile Facebook post. Vaccine.

[38] Sturm L, Kasting ML, Head KJ, Hartsock JA, Zimet GD (2021). Influenza vaccination in the time of COVID-19: A national U.S. survey of adults. Vaccine.

[39] Gandomi A, Haider M (2015). Beyond the hype: Big data concepts, methods, and analytics. International Journal of Information Management.

[40] Secinaro S, Calandra D, Secinaro A, Muthurangu V, Biancone P (2021). The role of artificial intelligence in healthcare: a structured literature review. BMC Med Inform Decis Mak.

[41] Schraeder TL (2019). Physician Communication Connecting with Patients, Peers, and the Public.

[42] Benis A, Barak Barkan R, Sela T, Harel N (2020). Communication Behavior Changes Between Patients With Diabetes and Healthcare Providers Over 9 Years: Retrospective Cohort Study. J Med Internet Res.

[43] Dai X, Bikdash M, Meyer B (2017). From social media to public health surveillance: Word embedding based clustering method for twitter classification.

[44] Henrich NJ (2011). Increasing pandemic vaccination rates with effective communication. Hum Vaccin.

[45] Feemster KA (2020). Building vaccine acceptance through communication and advocacy. Hum Vaccin Immunother.

[46] Sinclair J, Cardew-Hall M (2007). The folksonomy tag cloud: when is it useful?. Journal of Information Science.

[47] Robu V, Halpin H, Shepherd H (2009). Emergence of consensus and shared vocabularies in collaborative tagging systems. ACM Trans. Web.

[48] Wetzker R, Zimmermann C, Bauckhage C, Albayrak S (2010). I tag, you tag: translating tags for advanced user models. WSDM '10: Proceedings of the Third ACM International Conference on Web Search and Data Mining.

[49] Hönings H, Knapp D, Nguyễn BC, Richter D, Williams K, Dorsch I, Fietkiewicz KJ (2021). Health information diffusion on Twitter: The content and design of WHO tweets matter. Health Info Libr J.

[50] Sacco G (2000). Dynamic taxonomies: a model for large information bases. IEEE Trans. Knowl. Data Eng.

[51] Twitter API. Twitter Developer Platform.

[52] Zanin M, Aitya NAA, Basilio J, Baumbach J, Benis A, Behera CK, Bucholc M, Castiglione F, Chouvarda I, Comte B, Dao T, Ding X, Pujos-Guillot E, Filipovic N, Finn DP, Glass DH, Harel N, Iesmantas T, Ivanoska I, Joshi A, Boudjeltia KZ, Kaoui B, Kaur D, Maguire LP, McClean PL, McCombe N, de Miranda JL, Moisescu MA, Pappalardo F, Polster A, Prasad G, Rozman D, Sacala I, Sanchez-Bornot JM, Schmid JA, Sharp T, Solé-Casals J, Spiwok V, Spyrou GM, Stalidzans E, Stres B, Sustersic T, Symeonidis I, Tieri P, Todd S, Van Steen K, Veneva M, Wang D, Wang H, Wang H, Watterson S, Wong-Lin K, Yang S, Zou X, Schmidt HHHW (2021). An Early Stage Researcher's Primer on Systems Medicine Terminology. Netw Syst Med.

[53] Russell S, Norvig P (2020). Artificial Intelligence: A Modern Approach.

[54] Straus J, Kaufman L, Stern T (2014). The Blue Book of Grammar and Punctuation: An Easy-to-Use Guide with Clear Rules, Real-World Examples, and Reproducible Quizzes.

[55] Bird S, Klein E, Loper E (2009). Natural Language Processing with Python: Analyzing Text with the Natural Language Toolkit.

[56] Bergmanis T, Goldwater S (2018). Context Sensitive Neural Lemmatization with Lematus. Proceedings of the 2018 Conference of the North American Chapter of the Association for Computational Linguistics: Human Language Technologies, Volume 1 (Long Papers).

[57] Aggarwal C, Reddy C (2014). Data Clustering: Algorithms and Applications.

[58] Keogh E, Mueen A, Sammut C, Webb GI (2017). Curse of Dimensionality. Encyclopedia of Machine Learning and Data Mining.

[59] Vlachos M, Sammut C, Webb GI (2017). Dimensionality Reduction. Encyclopedia of Machine Learning and Data Mining.

[60] Mikolov T, Chen K, Corrado G, Dean J (2013). Efficient Estimation of Word Representations in Vector Space. arXiv.

[61] gensim 4.1.2. Python Package Index (PyPI).

[62] Butnaru A, Ionescu R (2017). From Image to Text Classification: A Novel Approach based on Clustering Word Embeddings. Procedia Computer Science.

[63] Li B, Drozd A, Guo Y, Liu T, Matsuoka S, Du X (2019). Scaling Word2Vec on Big Corpus. Data Sci. Eng.

[64] Rousseeuw PJ (1987). Silhouettes: A graphical aid to the interpretation and validation of cluster analysis. Journal of Computational and Applied Mathematics.

[65] van der Maaten L, Hinton G (2008). Visualizing Data using t-SNE. Journal of Machine Learning Research.

[66] Google Trends.

[67] Matschinske J, Alcaraz N, Benis A, Golebiewski M, Grimm DG, Heumos L, Kacprowski T, Lazareva O, List M, Louadi Z, Pauling JK, Pfeifer N, Röttger R, Schwämmle V, Sturm G, Traverso A, Van Steen K, de Freitas MV, Villalba Silva GC, Wee L, Wenke NK, Zanin M, Zolotareva O, Baumbach J, Blumenthal DB (2021). The AIMe registry for artificial intelligence in biomedical research. Nat Methods.

[68] Hoffman BL, Felter EM, Chu K, Shensa A, Hermann C, Wolynn T, Williams D, Primack BA (2019). It's not all about autism: The emerging landscape of anti-vaccination sentiment on Facebook. Vaccine.

[69] Burki T (2019). Vaccine misinformation and social media. The Lancet Digital Health.

[70] Ahmed I (2021). Dismantling the anti-vaxx industry. Nat Med.

[71] Lopreite M, Panzarasa P, Puliga M, Riccaboni M (2021). Early warnings of COVID-19 outbreaks across Europe from social media. Sci Rep.

[72] Weekly U.S. Influenza Surveillance Report. Centers for Disease Control and Prevention.

[73] Uyeki TM, Wentworth DE, Jernigan DB (2021). Influenza Activity in the US During the 2020-2021 Season. JAMA.

[74] Selecting the number of clusters with silhouette analysis on KMeans clustering. Scikit-learn developers.

[75] NLTK 3.6 documentation. NLTK.

[76] Capó M, Pérez A, Lozano JA (2017). An efficient approximation to the K-means clustering for massive data. Knowledge-Based Systems.

[77] Škrlj B, Kralj J, Lavrač N (2020). Embedding-based Silhouette community detection. Mach Learn.

[78] Lovmar L, Ahlford A, Jonsson M, Syvänen AC (2005). Silhouette scores for assessment of SNP genotype clusters. BMC Genomics.

[79] Maugeri A, Barchitta M, Agodi A (2020). A Clustering Approach to Classify Italian Regions and Provinces Based on Prevalence and Trend of SARS-CoV-2 Cases. Int J Environ Res Public Health.

[80] Ray S, Turi RH (1999). Determination of number of clusters in K-means clustering and application in colour segmentation.

[81] Thorndike RL (1953). Who belongs in the family?. Psychometrika.

[82] (2020). Pfizer and BioNTech announce vaccine candidate against COVID-19 achieved success in first interim analysis from phase 3 study. Pfizer.

[83] (2021). U.S. administers 293.7 mln doses of COVID-19 vaccines -CDC. Reuters.

[84] pytrends 4.7.3. Python Package Index (PyPI).

[85] Reporting COVID-19 Vaccinations in the United States. Centers for Disease Control and Prevention.

[86] Dong E, Du H, Gardner L (2020). An interactive web-based dashboard to track COVID-19 in real time. The Lancet Infectious Diseases.

[87] Baumgaertner B, Carlisle JE, Justwan F (2018). The influence of political ideology and trust on willingness to vaccinate. PLoS One.

[88] Debus M, Tosun J (2021). Political ideology and vaccination willingness: implications for policy design. Policy Sci.

[89] Allcott H, Boxell L, Conway J, Gentzkow M, Thaler M, Yang D (2020). Polarization and public health: Partisan differences in social distancing during the coronavirus pandemic. J Public Econ.

[90] Servick K (2021). COVID-19 measures also suppress flu-for now. Science.

[91] Feng L, Zhang T, Wang Q, Xie Y, Peng Z, Zheng J, Qin Y, Zhang M, Lai S, Wang D, Feng Z, Li Z, Gao GF (2021). Impact of COVID-19 outbreaks and interventions on influenza in China and the United States. Nat Commun.

[92] Razai M, Doerholt K, Ladhani S, Oakeshott P (2020). Coronavirus disease 2019 (covid-19): a guide for UK GPs. BMJ.

[93] Bagus P, Peña-Ramos JA, Sánchez-Bayón A (2021). COVID-19 and the Political Economy of Mass Hysteria. Int J Environ Res Public Health.

[94] Ortiz-Sánchez E, Velando-Soriano A, Pradas-Hernández L, Vargas-Román K, Gómez-Urquiza JL, Cañadas-De la Fuente GA, Albendín-García L (2020). Analysis of the Anti-Vaccine Movement in Social Networks: A Systematic Review. Int J Environ Res Public Health.

[95] (2015). US 2015-0235246 A1 - Cross-channel audience segmentation. Patent Center.

[96] Wendling C, Radisch J, Jacobzone S (2013). The Use of Social Media in Risk and Crisis Communication. OECD Working Papers on Public Governance.

[97] Freberg K, Palenchar MJ, Veil SR (2013). Managing and sharing H1N1 crisis information using social media bookmarking services. Public Relations Review.

[98] Guidry JP, Jin Y, Orr CA, Messner M, Meganck S (2017). Ebola on Instagram and Twitter: How health organizations address the health crisis in their social media engagement. Public Relations Review.

[99] Ozawa S, Clark S, Portnoy A, Grewal S, Stack ML, Sinha A, Mirelman A, Franklin H, Friberg IK, Tam Y, Walker N, Clark A, Ferrari M, Suraratdecha C, Sweet S, Goldie SJ, Garske T, Li M, Hansen PM, Johnson HL, Walker D (2017). Estimated economic impact of vaccinations in 73 low- and middle-income countries, 2001-2020. Bull World Health Organ.

[100] Selected social characteristics in the United States. United States Census Bureau.

[101] Xie X, Liang Y, Li X, Tan W (2019). CuLDA: Solving Large-scale LDA Problems on GPUs. HPDC '19: Proceedings of the 28th International Symposium on High-Performance Parallel and Distributed Computing.

[102] Kwok SWH, Vadde SK, Wang G (2021). Tweet Topics and Sentiments Relating to COVID-19 Vaccination Among Australian Twitter Users: Machine Learning Analysis. J Med Internet Res.

[103] Xue J, Chen J, Hu R, Chen C, Zheng C, Su Y, Zhu T (2020). Twitter Discussions and Emotions About the COVID-19 Pandemic: Machine Learning Approach. J Med Internet Res.

[104] (2015). US-20150088636-A1 - Classification of Geographic Performance Data. Patent Center.

[105] (2014). US 2014-0236715 A1 - Targeted Advertising in Social Media Networks. Patent Center.

[106] Rufai S, Bunce C (2020). World leaders' usage of Twitter in response to the COVID-19 pandemic: a content analysis. J Public Health (Oxf).

[107] Read W, Robertson N, McQuilken L, Ferdous A (2019). Consumer engagement on Twitter: perceptions of the brand matter. EJM.

[108] Dyer J, Kolic B (2020). Public risk perception and emotion on Twitter during the Covid-19 pandemic. Appl Netw Sci.

[109] Saleh SN, Lehmann CU, McDonald SA, Basit MA, Medford RJ (2021). Understanding public perception of coronavirus disease 2019 (COVID-19) social distancing on Twitter. Infect Control Hosp Epidemiol.

[110] Benis A, Tamburis O, Chronaki C, Moen A (2021). One Digital Health: A Unified Framework for Future Health Ecosystems. J Med Internet Res.

