# Supplementary material for: Change in Threads on Twitter Regarding Influenza, Vaccines, and Vaccination During the COVID-19 Pandemic: Artificial Intelligence–Based Infodemiology Study
Source: JMIR Infodemiology. 2021 Oct 14;1(1):e31983. doi: 10.2196/31983 (PMC8521455; doi:10.2196/31983)
Supplement: Multimedia Appendix 1 [file infodemiology_v1i1e31983_app1.pdf]

Multimedia Appendix 1: Number of tweets by month comprising at least one of the terms "flu," "vaccination," "vaccine," "vaxx," and "covid.".

**Month/Year**	**Number of tweets** (N=2,782,720) N (%)	**flu** (N=647,839) N (%)	**vaccination** (N=220,122) N (%)	**vaccine** (N=1,826,094) N (%)	**vaxx** (N=34,522) N (%)	**covid** (N=775,829) N (%)
12/2019	1,197 (0.04%)	631 (52.72%)	102 (8.52%)	400 (33.42%)	27 (2.26%)	0 (0.00%)
01/2020	43,074 (1.55%)	21,641 (50.24%)	2,702 (6.27%)	15,872 (36.85%)	1,403 (3.26%)	3,132 (7.27%)
02/2020	65,993 (2.37%)	36,387 (55.14%)	2,727 (4.13%)	21,729 (32.93%)	1,744 (2.64%)	15,130 (22.93%)
03/2020	238,675 (8.58%)	148,965 (62.41%)	4,497 (1.88%)	64,952 (27.21%)	1,665 (0.70%)	61,223 (25.65%)
04/2020	197,011 (7.08%)	78,394 (39.79%)	8,302 (4.21%)	102,358 (51.96%)	1,938 (0.98%)	45,300 (22.99%)
05/2020	150,584 (5.41%)	50,496 (33.53%)	5,875 (3.90%)	89,465 (59.41%)	1,853 (1.23%)	37,727 (25.05%)
06/2020	76,073 (2.73%)	28,289 (37.19%)	3,173 (4.17%)	40,463 (53.19%)	887 (1.17%)	18,932 (24.89%)
07/2020	128,239 (4.61%)	43,161 (33.66%)	4,891 (3.81%)	75,732 (59.06%)	1,537 (1.20%)	35,395 (27.60%)
08/2020	110,794 (3.98%)	34,001 (30.69%)	4,934 (4.45%)	69,554 (62.78%)	1,507 (1.36%)	30,633 (27.65%)
09/2020	125,957 (4.53%)	38,466 (30.54%)	4,106 (3.26%)	81,735 (64.89%)	1,906 (1.51%)	35,993 (28.58%)
10/2020	107,615 (3.87%)	47,340 (43.99%)	3,430 (3.19%)	53,833 (50.02%)	883 (0.82%)	32,817 (30.49%)
11/2020	162,202 (5.83%)	29,688 (18.30%)	6,292 (3.88%)	122,168 (75.32%)	1,563 (0.96%)	47,654 (29.38%)
2020/12	346,928 (12.47%)	34,709 (10.00%)	24,141 (6.96%)	281,748 (81.21%)	4,247 (1.22%)	108,525 (31.28%)
2021/01	227,599 (8.18%)	15,134 (6.65%)	31,640 (13.90%)	175,760 (77.22%)	2,010 (0.88%)	75,997 (33.39%)
02/2021	198,355 (7.13%)	11,830 (5.96%)	29,680 (14.96%)	151,984 (76.62%)	1,962 (0.99%)	67,374 (33.97%)
03/2021	291,140 (10.46%)	14,183 (4.87%)	42,204 (14.50%)	228,382 (78.44%)	3,683 (1.27%)	82,158 (28.22%)
04/2021	311,284 (11.19%)	14,524 (4.67%)	41,426 (13.31%)	249,959 (80.30%)	5,707 (1.83%)	77,839 (25.01%)
