# Supplementary material for: Change in Threads on Twitter Regarding Influenza, Vaccines, and Vaccination During the COVID-19 Pandemic: Artificial Intelligence–Based Infodemiology Study
Source: JMIR Infodemiology. 2021 Oct 14;1(1):e31983. doi: 10.2196/31983 (PMC8521455; doi:10.2196/31983)
Supplement: Multimedia Appendix 2 [file infodemiology_v1i1e31983_app2.pdf]

Multimedia Appendix 2: Optimal number of clusters, the related maximal silhouette score for each month, and the parameters used for creating the topic clustering.

Year	Month	Minimum term occurrence for being included in the analysis	Maximum distance between two words of a tweet for considering their potential semantic link	Number of features (words) investigated of the weekly tweets corpus	Silhouette Coefficient for the monthly optimal model with *k* clusters
2019	December	6	7	800	0.723
2020	January	3	7	800	0.642
	February	4	7	700	0.586
	March	3	7	600	0.650
	April	3	7	800	0.668
	May	3	7	700	0.667
	June	4	7	800	0.580
	July	3	6	500	0.663
	August	3	7	800	0.645
	September	3	7	700	0.663
	October	3	7	700	0.640
	November	3	7	800	0.667
	December	3	7	500	0.675
2021	January	3	7	700	0.717
	February	3	7	600	0.721
	March	3	7	500	0.716
	April	3	7	500	0.656
Overall	--	5	14	800	0.716
