# Supplementary material for: Change in Threads on Twitter Regarding Influenza, Vaccines, and Vaccination During the COVID-19 Pandemic: Artificial Intelligence–Based Infodemiology Study
Source: JMIR Infodemiology. 2021 Oct 14;1(1):e31983. doi: 10.2196/31983 (PMC8521455; doi:10.2196/31983)
Supplement: Multimedia Appendix 3 [file infodemiology_v1i1e31983_app3.pdf]

Multimedia Appendix 3: List of the 1000 most frequent n-grams in the 3 clusters.

List of the 1,000 most frequent n-grams for cluster 1:

vaccine, covid, flu, get, people, say, go, take, virus, like, vaccination, one, know, us, need, make, would, shot, think, work, year, die, want, death, time, still, many, first, even, give, see, come, test, well, bad, good, also, thats, state, day, pandemic, new, right, use, could, way, thing, tell, kill, case, never, help, vaccinate, cant, today, million, much, back, season, start, look, really, mask, let, month, may, everyone, number, stop, keep, receive, country, already, find, plan, sick, mean, believe, life, yet, week, wait, pfizer, end, happen, cause, nothing, world, yes, develop, show, live, report, please, every, wear_mask, child, since, talk, feel, kid, safe, without, hope, available, doctor, long, anyone, spanish, put, care, spread, cdc, actually, effective, enough, lot, great, ever, school, risk, far, ask, maybe, last, sure, open, ive, point, another, less, something, fact, around, news, two, read, next, dos, theyre, hospital, disease, ill, protect, real, science, anything, seem, cure, create, soon, distribution, someone, cold, research, symptom, might, person, trial, part, second, least, appointment, data, health, reason, question, high, include, probably, study, must, control, continue, family, hear, problem, deadly, others, close, require, scientist, moderna, issue, etc, likely, either, side_effect, place, patient, old, able, understand, different, dose, every_year, allow, treatment, due, ready, population, fauci, best, post, always, sign, ppl, change, company, public, expect, guess, fight, wrong, thanks, begin, little, worry, infect, home, claim, hard, whats, early, true, compare, seasonal, learn, administer, rollout, yeah, rate, agree, shut, possible, dead, body, wonder, fear, update, check, base, follow, catch, distribute, normal, result, important, low, human, id, influenza, infection, prevent, concern, chance, lockdown, herd_immunity, late, ok, matter, lead, age, become, theres, though, expert, idea, provide, share, pneumonia, schedule, medical, mrna, offer, dangerous, immunity, information, hold, community, approve, strain, vaxx, move, folk, full, via, group, roll, consider, mandate, development, teacher, release, common, fall, course, travel, response, hour, exactly, story, especially, fine, drug, literally, hit, yesterday, prove, death_rate, deal, bc, last_year, parent, supply, effort, set, answer, weve, past, treat, stay_home, uk, safety, increase, list, regular, healthy, whole, article, thousand, system, kind, rush, unless, produce, elderly, half, history, record, variant, fda, explain, stay, cannot, serious, miss, choice, outbreak, access, total, add, drive, pass, h1n1, current, administration, info, positive, panic, site, 2nd, event, county, plus, eligible, 1st, nurse, count, illness, realize, antibody, track, cancer, global, fail, seriously, easy, quarantine, polio, official, effect, reaction, march, program, mom, go_away, link, young, fast, process, several, however, choose, adult, level, year_old, zero, along, phase, actual, experience, immune_system, clear, mention, type, potential, nobody, cover, contract, exist, market, accord, absolutely, deliver, wish, medicine, recover, top, definitely, suppose, figure, ahead, hopefully, survive, slow, mortality_rate, similar, make_sure, area, quickly, none, imagine, future, currently, dr_fauci, demand, vaccinated, wear, drop, except, expose, thought, affect, cost, decide, focus, daily, lock, suggest, three, healthcare, among, staff, step, whether, anyway, avoid, mass, efficacy, evidence, hop, glad, nearly, handle, pretty, local, january, rest, worker, experimental, prepare, save_life, contagious, difference, date, large, proof, worldwide, complete, year_ago, first_dose, summer, reach, anti_vaxxer, appear, entire, saw, race, individual, student, shoot, common_cold, test_positive, opinion, theyve, small, despite, warn, high_risk, multiple, assume, later, responsible, mo, huge, completely, ignore, period, okay, senior, last_week, delay, source, correct, regard, accept, epidemic, testing, strong, benefit, recommend, alone, non, relate, encourage, united_state, survival_rate, certain, outside, interest, protection, second_dose, basically, make_sense, decade, average, impact, major, confirm, husband, speed, return, heres, discuss, jab, contain, policy, per, apparently, winter, vulnerable, grow, rather, colorado, worth, public_health, cv, immune, involve, decision, national, rise, hiv, meet, admit, action, bit, priority, mutation, remain, doubt, vaccines, form, target, statement, simply, estimate, suffer, lack, smart, social_distancing, clinical_trial, near, nope, lab, simple, single, mine, option, website, heard, store, build, good_news, reduce, production, longer, fever, sars, willing, annual, even_though, situation, goal, solution, otherwise, note, healthcare_worker, mutate, perhaps, itll, forever, disappear, society, harm, address, necessary, clearly, honestly, daughter, online, every_day, weird, till, carry, approval, obviously, damage, ridiculous, safe_effective, reality, ebola, totally, sound, super, severe, argument, hospitalization, long_term, researcher, injury, notice, within, amount, progress, location, mild, med, manufacture, threat, personally, quick, short, measure, usually, quite, yearly, truly, therapeutic, service, negative, immediately, term, four, technology, majority, message, injection, detail, unfortunately, scary, condition, opportunity, possibly, contact, effectiveness, reopen, percent, certainly, europe, prior, ensure, approach, emergency, stats, twice, restriction, challenge, present, convince, blood_clot, pfizer_moderna, recently, novel, occur, waste, hospitalize, massive, decline, often, depend, guarantee, mark, key, mostly, failure, recent, big_deal, wash_hand, doc, horrible, eventually, fully_vaccinate, cell, surge, animal, danger, spike, fully, measles, misinformation, design, resource, comparison, delivery, strategy, timeline, educate, extra, neither, manage, medication, general, regardless, spring, couple, aware, pause, felt, shutdown, vs, sars_cov, limit, moment, successful, across, thread, hcq, health_care_worker, product, shingle, wed, terrible, interesting, false, beyond, yr, prioritize, argue, double, ive_never, success, everywhere, view, sort, excite, refer, curious, autism, back_normal, respond, dna, two_week, month_ago, statistic, faster, pop, amaze, hesitancy, nursing_home,

education, feeling, specific, stop_spread, cough, manufacturer, critical, although, wave, recovery, practice, enter, grateful, third, plenty, discover, thru, allergy, math, hundred, paper, account, advocate, facility, unknown, gt, rna, interested, asap, worried, ex_le, fair, transmission, predict, downplay, oxford, qualify, previous, blood, determine, hpv, project, weak, difficult, ventilator, provider, death_toll, around_world, standard, seek, advice, mortality, federal_government, requirement, improve, ingredient, sometimes, italy, guideline, additional, immunization, version, struggle, model, mask_mandate, widespread, perfect, apply, lucky, health_care, booster, complication, essential_worker, availability, eradicate, eliminate, tho, typical, review, headline, everyone_else, employer, teach, confuse, logic, week_ago, essential, easily, position, extremely, ability, 2nd_dose, round, factor, respect, c19, precaution, infected, pain, accurate, new_strain, careful, mass_vaccination, common_sense, purpose, identify, june, become_available, useless, asymptomatic, andor, idk, sooner, usual, unlike, nyc, shortage, theory, repeat, oppose, perspective, physician, hysteria, exposure, impossible, personal, boost, er, reminder, beginning, tool, mn, status, self, besides, isolate, natural, look_forward, inform, bother, subject, experiment, proven, living, az, pretty_sure, overall, therefore, suspect, long_term_effect, safely, directly, smallpox, search, finish, associate, discussion, implement, earlier, next_month, knowledge, globally, chart, diagnose, initial, trend, conversation, viral, placebo, fatality_rate, normally, thankful, label, stay_safe, obvious, combat, tech, sadly, stage, family_member, peak, ton, rare, combine, honest, pre, protocol, fda_approve, nurse_home, request, distance, disagree, appreciate, appt, direct, vitamin, awful, lung, skip, significant, lethal, fast_track, sickness, value, insurance, potentially, social_distance, answer_question, wo, annually, cheap, test_negative, basic, trouble, percentage, document, advise, sense, hesitant

List of the 1,000 most frequent n-grams for cluster 2:
clinic, johnson_johnson, dr, tomorrow, april, announce, team, appointments_longer_available_walgreens, volunteer, morning, visit, astrazeneca, resident, covidvaccine, employee, next_week, friday, monday, missouri, pharmacy, zip_code, register, center, candidate, join, pfizers, tuesday, iowa, million_dos, november, arrive, california, thursday, december, minnesota, weekend, wednesday, host, federal, co, partner, october, member, february, vaccination_site, jan, feb, tonight, interview, oklahoma, five, appointment_available_walgreens_saint, kansa, saint_louis, saturday, utah, expand, urge, email, wisconsin, launch, modernas, college, health_department, eligibility, university, health_official, hi, sunday, shipment, page, july, new_york_time, participate, pfizer_biontech, dec, announcement, covid19vaccine, distribution_plan, ceo, nov, amid, agency, appointments_longer_available_walmart, kansas_city, smartnews, arkansas, pharmacist, va, pm, final, chief, september, nebraska, appointment_available, former, reveal, apr_apr_sign_zip, fact_check, yay, episode, veteran, md, louis_apr_apr_sign, meeting, task_force, aim, tip, alert, director, spain, h1n1_swine_flu, st_louis, six, series, michigan, district, survey, educator, vaccineswork, schedule_appointment, nih, receive_first_dose, emergency_use, august, tennessee, brazil, frontline_worker, human_trial, excited, yahoo, resume, sign_zip_code, walgreens, upcoming, authorize, high_school, submit, mexico, first_round, weekly, officially, dy, app, washington_post, 3rd, appointment_available_walgreens_kansas, pet, novavax, ohio, announces, equity, letter, first_responder, investigation, dr_anthony_fauci, oct, brief, assist, covid_socialdistancing_flattenthecurve, gatewaypundit, registration, press_conference, illinois, drive_thru, washington, join_us, podcast, partnership, mass_vaccination_site, health_worker, click, britain, apr_sign_zip_code, icymi, denver, tracker, chicago, astrazenecas, ago, health_expert, guide, congratulation, world_health_organization, afternoon, sputnik, text, france, la, nbcnews, department, wearamask, live_update, googlenews, getvaccinated, johnson, break_news, feature, south_dakota, tn, conduct, birthday, day_later, suspend, thank_president, investor, panel, nypost, arizona, pediatrician, tune, breakthrough, unit, phase_1b, wait_list, virtual, dos_administer, via_nyt, new_mexico, professor, city_apr_apr_sign, south, exclusive, british, age_old, john, nyt, seattle, developer, agreement, appointments_longer_available_cvs, louisiana, wsj, israeli, minnesotan, authorization, lawmaker, cdc_fda, teen, mar, european, stl, england, yahoonews, hhs, tour, thanksgiving, coronavirusvaccine, usatoday, shortly, coronaoutbreak, pledge, committee, vaccination_aign, underway, flushot, mobile, south_africa, statewide, across_state, initiative, 50k, soon_possible, notify, file, st, hubby, reuters, firm, mid, cuba, memphis, affordable, nevada, cruise, cdc_director, appointment_available_hy_vee, shelby_county, merck, wi, msnbc, bloomberg, kansas, business_insider, approve_fda, 10k, cnbc, fantastic, food_drug_administration, politico, surpass, operationwarpspeed, warns, north, officer, school_district, missourian, mass_vaccination_event, pfizerbiontech, roll_sleeve, npr, virginia, seven, historic, summit, hub, ct, maskup, massachusetts, coronaoutbreak_wuhanflu_chinaflu, innovation, virus_coronavirusoutbreak_coronaviruswuhan_2019ncov, dolly_parton, urgent, session, eligible_receive, prof, maryland, mailonline, reportedly, institute, 1b, briefing, rank, discovery, award, next_phase, arrival, president_donald_trump, postpone, oklahoman, first_dose_pfizer, guest, read_enmnewshealth, bbc_news, center_disease_control_prevention, childrens, central, arm_sore, health_dept, conference, truck_news, biontech, sept, webinar, coloradan, david, al, kc, oregon, gear, publichealth, aag_aag2020, thrill, 4th, top_story, fda_cdc, jj, robert_kennedy_jr, 20k, slot, french, watch_video, louis_apr_sign_zip, tackle, scramble, expedite, may_sign_zip, pfe, journal, minister, cc, updated, polis, breitbartnews, coronaviruspandemic, light_end_tunnel, blog, health_center, dashboard,

executive, transportation_ship_logistics_follow, author, czar, urgent_care, prep, biotech, st_louis_county, coronavirusoutbreak, regulator, pakistan, chat, statnews, vaccinessavelives, italian, association, manager, president_joe_biden, centre, segment, receive_second_dose, school_staff, start_monday, question_answer, laboratory, petition, put_together, back_negative, percent_effective, pod, de, need_worry_whats, appointments_longer_available_hy, priority_group, explore, representative, pass_away, fresh, mass_vaccination_clinic, english, collaboration, eight, zero_hedge, 1m, london, inmate, kit, foxnews, ap, nine, medical_center, shots, billion_dos, african_american, reschedule, whole_family, mississippi, eager, covax, evening, lottery, broadcast, mega, oxford_astrazeneca, locate, frontline, proposal, arm_hurt, thx, paul, nigeria, offer_free, quarter, alabama, trail, minneapolis, deadline, japanese, milestone, per_week, press_release, hy_vee, covidvaccines, 100m, indiana, nfl, adviser, regional, dentist, australian, 19vaccine, portal, john_hopkins, next_step, forecast, childrens_health_defense, walz, medical_worker, pennsylvania, appointment_schedule, covid2019, ty, alliance, offering, ne, michael, mercy, joint, metro, nc, abc_news, pentagon, advisor, new_jersey, administrator, tap, dedicate, phd, ksleg, kudos, nursing_home_resident, local_pharmacy, todays, wear_face, jennings, vaccinator, houston, reveals, hotline, omaha, covidvaccination, commission, oxford_university, local_health_department, two_week_ago, commitment, 5k, boston, prospect, guardian, scotland, douglas_county, johnsonandjohnson, dm, ows, discus, anthony_fauci, finncil_red, phizer, spring_break, stayhome, scott, spoke, initiate, soar, public_health_expert, indias, zika, president_elect_joe_biden, il, attorney, dr_birx, log, allotment, alaska, county_health_department, research_development, mayo_clinic, friend_mine, nhs, newspaper, chair, bill_melinda_gate_foundation, outline, new_story_npr, bridge, virus_coronavirusoutbreak_covid2019_wuhanvirus, covaxin, nvax, split, state_health_official, utpol_utah, first_dose_moderna, philadelphia, town_hall, ned_resign, denmark, hour_later, steve, ms, oversee, across_globe, sec, huffpost, new_york_city, across_world, noon, ireland, norway, first_serve, ontario, cu, compensation, reservation, expert_warn, washington_state, restart, connector, inc, niece, visitor, teenager, ups, download, fun_fact, fluseason, presentation, column, adult_eligible, gtgt, outreach, appointments_longer_available_cv, parking_lot, appointment_available_walgreens_jennings, wyoming, college_student, dod, aug, coronavirususa, dispense, clinicaltrials, tribe, hall, pfizervaccine, exciting, missouris, become_eligible, tulsa, bbc, cv_walgreens, salk, notification, province, approve_covid2019, economic_recovery, biospace_lifesciences_biotechnology_pharmaceutical, sedgwick_county, gavi, apr, enrol, ar, provide_update, staff_member, philippine, kansan, surgeon_general, graduate, vice_president, cbs_news, keep_eye, kentucky, teacher_staff, coming_week, native, redfield, maine, health_service, wellness, program_engage_80k_patient, pre_register, drfauci, second_round, first_batch, reuters_via_nyt, mill, utahns, library, tunnel, pay_billion, nbc_news, socialdistancing, book_appointment, pharmacy_technician, coronavirususa_pandemic, end_october, phase_1a, janssen, one_step_closer, certify, news_conference, originate_spain, far_behind, start_kansa, james, los_angeles, slide, year_old_woman, epochtimes, top_priority, nursing, bell, vow, atlanta, mobilize, idaho, faq, inovio, brit, est, phase_clinical_trial, informative, part_operation_warp_speed, west_virginia, expansion, 5m, medtwitter, org, year_old_mother, rd, speaks, boris_johnson, hour_ago, documentary, midst, pending, big_threat, welp, aigns, collaborate, public_health_emergency, outlook, waitlist, infectious_disease_expert, draft, sinovac, highly_recommend, emergency_authorization, post_edit, show_promise, pilot, 12k, bishop, johnson_county, extra_dos, gratitude, harvard, ino, san_francisco, glad_hear, cooperation, midwest, presser, couple_week_ago, com, recruit, prominent, economist, connecticut, appointment_available_walgreens, kevin, ii, alongside, thehill, cbs, registry, et, frontline_healthcare_worker, atlantic, sen, fighter, identification, nbc, robert, gilead, russias, ph, commissioner, community_color, tipsnews, huffpostpol, abc, hawaii, smooth, andrew, council, ahead_schedule, labs, mdh, european_union, early_march, sa, peter, reader, stun, arrives, article_reuters, safety_effectiveness, hydroxycloroquine, gardasil, incoming, twin, first_shipment, via_nyt_new_york, relief_package, nearby, small_town, job_economy, arena, vatican, miami, click_link, emergency_approval, arkansan, pause_johnson_johnson, moleg, 3m, elementary_school, grandson, ia, spark, sanofi, rare_blood_clot, lake, stanford, beijing, mysterious, journey, adam, factsmatter, newsletter, tomorrow_morning, united, coronavaccine, coordination, testimony, staffer, bay, writes, speaker, verge, native_american, dept, southern, covid19vaccines, passenger, 5th, hesitate, edition, loom, around_globe, azn, earlier_week, gen, smith, kroger, doh, monday_morning, kcvaccinewatch, patch, hail, rival, ft, thisisourshot, uks, cvs, mum, next_summer, lincoln, earnings, mass_vaccination_sit, ricketts, ray, north_carolina, sat, apt, utahs, ps5, employer_require, rev, lifepetitions_petition_culture_life, prepares, jeff, mary, firefighter, news_lifesite, declare_national_emergency, black_jack, cause_brain_damage, health_agency, downtown, since_october, critical_care, historian, breakingnews, fourth, counting, without_fire, nebraskan, jonas_salk, taiwan, additional_dos, sugar_cube, commentary, coronavirusupdates, fridge, h3n2, kelly, web, san_diego, editorial, trading, upgrade, spread_across, fightflu, onsite, world_1st_inactivated, okc, newly, risk_educationelevation_kidney_patient, free_charge, quest, part_warp_speed, health_minister, subscribe, whats_difference, preparedness, fedex, ibio, 2b, renew, conquer, mass_murder_american, calendar, dr_faucis, testify, full_story, excitement, virus_republican_act, remove_million_infected, laura, pave_way, sport_event, secretary, safety_concern, cancellation, publication, shot_retweet,

community_member, wv, detroit, phase_iii, halloween, waiver, vega, impressed, chile, coronavirusupdate, spokesperson, surgeon, brace, health_emergency, state_department_health, unapproved, across_nation, extension, indigenous, mlb, programme, ave, dry_ice, health_care_professional, second_dose_moderna, independence, stltoday, case_hospitalization_death, designate, 1k, vaccination_programme, saint_paul, springfield, midnight, move_phase, city_apr_sign_zip, philly, sep_pm_et, futuredanger_indicator_vaccination, cuban, spur, magazine, analyst, bolster, apps, appointment_available_walgreens_black, navajo_nation, scheduling, department_health, colorados, lee, businessinsider, appointment_available_walmart_kansas, adolescent, effect_expose_heatmap, wall_street_journal, dallas, south_african, president_elect_biden, se, abc_news_abc, mom_dad, set_aside, oxfordastrazeneca, brazilian, iowas, thedailybeast, fluvaccine, right_around_corner, jack_apr_apr_sign, admits, national_institute_health, mother_law

List of the 1,000 most frequent n-grams for cluster 3:
trump, american, call, try, china, lie, swine, government, biden, trust, push, force, bill_gate, big, free, money, president, america, refuse, watch, thank, remember, fuck, medium, mandatory, line, support, everything, love, pay, man, run, job, lol, leave, turn, shit, lose, passport, guy, oh, gonna, order, hell, election, chinese, away, friend, hoax, vote, save, bring, stupid, instead, name, business, democrat, gate, send, kung_flu, play, usa, anti, listen, buy, wuhan, yall, tweet, economy, blame, word, fake, damn, arm, break, head, governor, youtube, sorry, god, truth, hand, billion, happy, forget, obama, hey, sell, office, idiot, republican, face, finally, fund, citizen, promise, india, nation, leader, woman, video, wake, crazy, racist, black, inject, city, stand, speak, game, bill, credit, win, baby, scar, sit, big_pharma, hate, political, govt, president_trump, military, mind, politician, stuff, power, dems, together, minute, act, whatever, sad, hurt, profit, bet, aid, freedom, theyll, behind, afraid, charge, florida, code, wow, hes, wife, joke, russia, deny, bullshit, comment, promote, dumb, operation_warp_speed, funny, stock, house, card, destroy, trump_administration, white_house, attack, gop, twitter, walk, sound_like, nice, spend, anymore, israel, eat, party, rule, poor, side, evil, write, law, cnn, surprise, fire, suck, food, beat, patent, eu, dad, gov, book, bird, son, pray, agenda, war, throw, warp_speed, cancel, crisis, white, aint, chip, cut, liberal, btw, politics, ya, texas, take_credit, left, ny, deserve, crap, msm, brain, ur, everybody, stick, poison, propaganda, joe, block, mess, donald_trump, hide, remove, cant_wait, dude, leadership, wtf, freak, russian, ban, fool, meanwhile, congress, yep, anti_vaxx, pick, new_york, rich, ship, mother, fix, kick, africa, screw, canada, fly, pro, scam, conspiracy, moron, debate, potus, else, fun, hydroxychloroquine, funding, pharma, bed, microchip, cool, wanna, fucking, enjoy, excuse, ignorant, joe_biden, attempt, special, pull, inside, blue, miracle, rid, sleep, fill, relief, act_like, cuomo, car, shame, cry, sheep, gotta, straight, fraud, conspiracy_theory, fake_news, corrupt, bidens, ppe, tv, nonsense, class, germany, eye, night, insane, bug, plague, lmao, tire, dog, bunch, everyday, narrative, criminal, industry, quit, bro, maker, steal, church, protest, first_line, trumps, putin, liar, front, serve, abortion, good_luck, christmas, remind, fault, rally, jump, light, complain, penny, google, 5g, crowd, scare, somehow, amazing, earth, boy, proud, omg, anyone_else, asian, kinda, rest_us, welcome, room, water, needle, hype, stomach, touch, elect, maga, guinea_pig, pretend, police, bear, picture, anybody, girl, sister, bitch, cuz, poll, fan, mr, stimulus, hong_kong, social_medium, air, raise, press, heart, price, conservative, celebrate, facebook, invest, brother, racism, disaster, victim, stupidity, humanity, shock, vaccination_card, mark_beast, sue, mad, busy, fed, cause_autism, front_line, drink, investigate, pharmaceutical_company, awesome, neighbor, gun, clean, last_night, phone, bar, murder, battle, suddenly, ccp, aign, father, ignorance, selfish, wheres, sport, huh, reject, sir, lady, rona, supporter, alive, hero, town, pas, record_time, hang, men, purchase, organization, jesus, spot, piece, smh, whos, board, laugh, biden_administration, threaten, feed, wh, fox_news, piss, americans, invent, faith, court, secret, favor, follower, secure, life_save, wipe, bs, restaurant, tax, yea, thank_god, aka, attention, declare, train, tie, whose, photo, trump_supporter, killer, blow, president_biden, planet, authority, voter, deep_state, burn, movie, quote, defeat, disgust, clue, plandemic, indian, untested, politicize, arrest, pay_attention, stock_market, depopulation, defend, border, fear_monger, street, trip, asshole, desantis, ruin, donate, insist, wonderful, prison, dear, yo, scream, senator, internet, building, reporter, jail, nah, buddy, lay, admin, pathetic, trump_admin, calm, trumpvirus, attend, would_rather, coincidence, connect, elite, upset, platform, somebody, hat, population_control, pig, middle, voice, global_pandemic, private, fox, praise, ad, til, clown, kill_thousand, legal, un, prayer, replace, publicly, vet, dream, fl, grandma, tout, angry, beg, walmart, comply, drug_company, chill, red, toxic, pack, worry_whats, hot, mouth, incompetence, favorite, nwo, shove, sudden, energy, christian, connection, silly, body_choice, operation, corruption, cult, small_business, trade, sale, vacation, criticize, shop, injure, hmmm, deep, garbage, hug, hypocrite, army, desperate, bat, cash, minority, color, sacrifice, hire, enforce, ugh, stockpile, abt, door, swear, great_job, oh_wait, billgates, illegal, absolute, heal, tds, infrastructure, unemployment, incompetent, enemy, reserve, international, network, german, haha, mayor, movement, customer, pharmaceutical, genius, dem, journalist, crime, judge, digital, strike, chaos, everyones, plant, bout, land, pill, lawsuit, climate_change, magic, park, nightmare, trash, rest_world, hmm, investment, tired, soldier, pump, grocery_store, dare, ride, leftist, outrage, nut, patriot, mr_president, um, beautiful, powerful, brand, hello, ground, stimulus_check, dollar, georgia, public_school, hack, box, beer, tell_truth, smoke, hed,

canadian, denier, privilege, organize, face_mask, resist, bank, liberty, influence, manipulate, corporation, wealthy, cat, king, em, call_hoax, got, divide, pa, pelosi, brag, fema, donald, commit, accuse, communist, disinformation, loser, disinfectant, heck, african, bos, scared, speech, brainwash, cop, resign, riot, whole_thing, computer, foot, constitution, tyranny, concert, con, fat, rip, suicide, senate, wall, legit, withhold, mock, dumbass, globalist, dc, table, invite, certificate, airline, b4, gym, conspiracy_theorist, consent, badly, whole_world, heat, rep, everything_else, flight, nj, west, religion, blind, empty, constituent, karen, called, honor, financial, pocket, nazi, uh, congrats, spin, pic, fee, star, plane, sun, billionaire, export, dark, insanity, weapon, denial, gate_foundation, unbelievable, homeless, kiss, ex, bless, oh_yeah, making, cousin, democratic, publix, harris, sham, assistance, republicans, commercial, trillion, immigrant, wash, hurry, jump_line, dirty, zombie, billion_dollar, pls, monster, national_guard, amazon, golf, genocide, accomplish, hypocrisy, trick, orange, cheer, hows, covidiots, old_age, image, sheeple, fb, security, stream, coordinate, duh, cross, stfu, creation, capitalism, bite, sight, draw, god_bless, nose, skin, suspicious, lately, democracy, change_mind, mail, soul, troll, tear, content, alot, fear_mongering, palestinian, lord, expire, crash, green, ffs, americas, bleach, ticket, switch, qanon, prisoner, destroy_economy, intentionally, worthless, rhetoric, ah, whoever, libs, ing, fellow, rock, ass, collapse, religious, socialist, wu, rant, presidency, foundation, foreign, meant, voting, gift, peace, psa, peep, accident, tiny, hilarious, troop, lose_job, knew, hole, ball, socialism, trump2020, rumor, machine, fuel, depression, crush, caign, shall, shitty, camera, entire_world, shirt, neighborhood, wait_till, career, fakenews, theater, pharma_company, hearing, iran, breath, giant, scheme, djt, change_dna, sex, retire, engineer, hoard, eugenics, mile, brilliant, monkey, whine, chinavirus, punish, stance, mf, censor, meme, traitor, terrorist, bubble, waste_time, red_state, silence, anti_science, truck, shameful, convenient, bottom, abuse, idiotic, factory, style, grip, bioweapon, po, rag, embarrass, sweet, bigpharma, reward, plot, trumpers, package, union, bully, bus, forced_vaccination, microsoft, obama_biden, desire, apologize, dump, loose, celebrity, kamala, endorse, drag, ha, vp, fell, beware, reverse, kamala_harris, collect, witness, absolutely_nothing, take_responsibility, club, surprised, pure, smell, shout, sterilize, era, lawyer, donation, hair, mike, better, idiots, globalists, transition, grandmother, wth, eh, blue_state, intention, undermine, impeachment, wind, chicken, rat, daddy, fuckin, grab, ai, brave, song, airport, obamas, dictator, yell, tag, tf
