# Supplementary material for: Change in Threads on Twitter Regarding Influenza, Vaccines, and Vaccination During the COVID-19 Pandemic: Artificial Intelligence–Based Infodemiology Study
Source: JMIR Infodemiology. 2021 Oct 14;1(1):e31983. doi: 10.2196/31983 (PMC8521455; doi:10.2196/31983)
Supplement: Multimedia Appendix 4 [file infodemiology_v1i1e31983_app4.pdf]

Multimedia Appendix 4: N-grams having the highest increase in occurrence week over week.

n	N-gram	Week starting on	Previous week n/N (%)	n/N (%)	Change Week over Week n (%)
1	vaccine	November 09, 2020	8,151/2,046,418 (0.40%)	50,987/2,046,418 (2.49%)	+42,836 (525.5%)
1	covid	February 24, 2020	1,988/865,901 (0.23%)	11,838/865,901 (1.37%)	+9,850 (495.5%)
1	flu	February 24, 2020	6,606/850,385 (0.78%)	26,603/850,385 (3.13%)	+19,997 (302.7%)
1	get	November 09, 2020	2,953/686,401 (0.43%)	10,398/686,401 (1.51%)	+7,445 (252.1%)
1	people	February 24, 2020	947/391,101 (0.24%)	6,789/391,101 (1.74%)	+5,842 (616.1%)
1	say	November 09, 2020	1,223/265,933 (0.46%)	7,436/265,933 (2.80%)	+6,213 (508.0%)
1	go	November 09, 2020	1,385/241,735 (0.57%)	5,462/241,735 (2.26%)	+4,077 (294.4%)
1	take	November 09, 2020	974/230,778 (0.42%)	7,110/230,778 (3.08%)	+6,136 (630.0%)
1	vaccination	November 09, 2020	537/228,432 (0.24%)	1,830/228,432 (0.80%)	+1,293 (240.1%)
1	virus	February 24, 2020	1,186/222,040 (0.53%)	5,366/222,040 (2.42%)	+4,180 (352.4%)
2	covid, vaccine	February 24, 2020	162/334,092 (0.05%)	1,549/334,092 (0.46%)	+1,387 (856.2%)
2	get, vaccine	November 09, 2020	302/149,333 (0.20%)	1,987/149,333 (1.33%)	+1,685 (557.9%)
2	flu, shot	February 24, 2020	407/68,518 (0.59%)	1,414/68,518 (2.06%)	+1,007 (247.4%)
2	take, vaccine	November 09, 2020	138/51,553 (0.27%)	1,207/51,553 (2.34%)	+1,069 (774.6%)
2	get, flu	February 24, 2020	487/50,962 (0.96%)	1,517/50,962 (2.98%)	+1,030 (211.5%)
2	get, covid	November 09, 2020	134/48,993 (0.27%)	486/48,993 (0.99%)	+352 (262.7%)
2	flu, season	February 24, 2020	401/47,727 (0.84%)	1,211/47,727 (2.54%)	+810 (202.0%)
2	wear, mask	November 09, 2020	288/46,062 (0.63%)	1,049/46,062 (2.28%)	+761 (264.2%)

2	swine, flu	February 24, 2020	73/41,231 (0.18%)	1,046/41,231 (2.54%)	+973 (1313.5%)
2	spanish, flu	February 24, 2020	114/36,555 (0.31%)	815/36,555 (2.23%)	+701 (614.9%)
3	get, covid, vaccine	November 30, 2020	272/33,108 (0.82%)	1,208/33,108 (3.65%)	+936 (344.1%)
3	get, flu, shot	February 24, 2020	178/25,909 (0.69%)	635/25,909 (2.45%)	+457 (256.7%)
3	vaccine, appointment, available	March 15, 2021	205/18,678 (1.10%)	748/18,678 (4.00%)	+543 (264.9%)
3	vaccine, appointments, longer	March 15, 2021	115/16,287 (0.71%)	543/16,287 (3.33%)	+428 (372.2%)
3	appointments, longer, available	March 15, 2021	115/16,287 (0.71%)	543/16,287 (3.33%)	+428 (372.2%)
3	sign, zip, code	March 15, 2021	114/16,035 (0.71%)	562/16,035 (3.50%)	+448 (393.0%)
3	apr, sign, zip	March 29, 2021	236/13,192 (1.79%)	2,834/13,192 (21.48%)	+2,598 (1095.8%)
3	appointment, available, walgreens	March 29, 2021	553/10,721 (5.16%)	2,549/10,721 (23.78%)	+1,996 (360.9%)
3	longer, available, walgreens	March 29, 2021	535/10,361 (5.16%)	2,753/10,361 (26.57%)	+2,218 (414.6%)
3	receive, covid, vaccine	December 14, 2020	370/9,275 (3.99%)	1,404/9,275 (15.14%)	+1,034 (279.5%)
4	vaccine, appointments, longer, available	March 15, 2021	115/16,287 (0.71%)	543/16,287 (3.33%)	+428 (372.2%)
4	apr, sign, zip, code	March 29, 2021	236/13,192 (1.79%)	2,834/13,192 (21.48%)	+2,598 (1095.8%)
4	vaccine, appointment, available, walgreens	March 29, 2021	551/10,719 (5.14%)	2,548/10,719 (23.77%)	+1,997 (361.6%)
4	appointments, longer, available, walgreens	March 29, 2021	535/10,361 (5.16%)	2,753/10,361 (26.57%)	+2,218 (414.6%)
4	apr, apr, sign, zip	March 29, 2021	742/8,523 (8.71%)	1,485/8,523 (17.42%)	+743 (100.0%)
4	available, walgreens, saint, louis	March 29, 2021	163/6,889 (2.37%)	2,157/6,889 (31.31%)	+1,994 (1223.3%)
4	sign, zip, code, vaccine	March 29, 2021	290/6,716 (4.32%)	1,026/6,716 (15.28%)	+736 (252.6%)
4	zip, code, vaccine, appointment	March 29, 2021	268/6,271 (4.27%)	945/6,271 (15.07%)	+677 (252.6%)

4	code, vaccine, appointment, available	March 29, 2021	268/6,264 (4.28%)	942/6,264 (15.04%)	+674 (251.5%)
4	longer, available, walgreens, saint	March 29, 2021	134/4,074 (3.29%)	1,221/4,074 (29.97%)	+1,087 (811.2%)
